# Genetic conflicts with *Plasmodium* parasites and functional constraints shape the evolution of erythrocyte cytoskeletal proteins

**DOI:** 10.1038/s41598-018-33049-y

**Published:** 2018-10-02

**Authors:** Manuela Sironi, Diego Forni, Mario Clerici, Rachele Cagliani

**Affiliations:** 1 Bioinformatics, Scientific Institute, IRCCS E. Medea, 23842 Bosisio Parini, Lecco Italy; 2 Department of Physiopathology and Transplantation, University of Milan, 20090 Milan, Italy; 3 Don C. Gnocchi Foundation ONLUS, IRCCS, 20148 Milan, Italy

## Abstract

*Plasmodium* parasites exerted a strong selective pressure on primate genomes and mutations in genes encoding erythrocyte cytoskeleton proteins (ECP) determine protective effects against *Plasmodium* infection/pathogenesis. We thus hypothesized that ECP-encoding genes have evolved in response to *Plasmodium*-driven selection. We analyzed the evolutionary history of 15 ECP-encoding genes in primates, as well as of their *Plasmodium*-encoded ligands (KAHRP, MESA and EMP3). Results indicated that *EPB42*, *SLC4A1*, and *SPTA1* evolved under pervasive positive selection and that episodes of positive selection tended to occur more frequently in primate species that host a larger number of *Plasmodium* parasites. Conversely, several genes, including *ANK1* and *SPTB*, displayed extensive signatures of purifying selection in primate phylogenies, Homininae lineages, and human populations, suggesting strong functional constraints. Analysis of *Plasmodium* genes indicated adaptive evolution in *MESA* and *KAHRP*; in the latter, different positively selected sites were located in the spectrin-binding domains. Because most of the positively selected sites in alpha-spectrin localized to the domains involved in the interaction with KAHRP, we suggest that the two proteins are engaged in an arms-race scenario. This observation is relevant because KAHRP is essential for the formation of “knobs”, which represent a major virulence determinant for *P*. *falciparum*.

## Introduction

Malaria is annually responsible for hundreds of thousands of deaths and millions of illnesses per year (WHO, http://www.who.int/malaria/publications/world-malaria-report-2016/). It is caused by protozoan parasites of the genus *Plasmodium*, and, although there are about 30 *Plasmodium* species that infect primates^1^, only five (*P*. *falciparum*, *P*. *vivax*, *P*. *malariae*, *P*. *knowlesi*, and *P*. *ovale*) cause malaria in humans. In particular, *P*. *falciparum* and *P*. *vivax* are the most prevalent, with *P*. *falciparum* causing the deadliest form of malaria (see review^2^).

The extreme virulence of *P*. *falciparum* and its propensity to cause severe disease symptoms are due to its ability to invade and refurbish mature human red blood cells (RBCs). In fact, during parasite asexual development, infected RBCs (iRBCs) undergo extensive phenotypic changes in their structure and function. Modification in erythrocyte surface topology, membrane permeability, stiffness, adhesiveness, and deformability lead to sequestration of iRBCs in the microvasculature preventing parasite splenic clearance and causing obstruction (reviewed in^3^).

*P*. *falciparum* achieves these changes in the iRBC structure by exporting parasite proteins into the erythrocytes. Some of these proteins interact with host components of the cytoskeleton and plasma membrane and lead to the formation of cytoadhesive and antigenic supramolecular protrusions (“knobs”) at the iRBC surface^3,4^. Knobs act as platforms for the presentation of *P*. *falciparum* erythrocyte membrane protein 1 (PfEMP1): this membrane-embedded protein is responsible for adhesion to the vascular endothelium^5^ and represents a major *P*. *falciparum* parasite virulence factor. PfEMP1 also interacts with *P*. *falciparum* KAHRP (Knob-associated Histidine-rich Protein), which in turn binds host ankyrin as well as spectrin α- and β-chains^6–8^. In addition to KAHRP, other exported *P*. *falciparum* proteins, namely erythrocyte membrane protein 3 (PfEMP3), mature parasite-infected erythrocyte surface antigen (MESA, also known as pfEMP2), *Plasmodium* helical interspersed subtelomeric (PHIST) proteins, and ring-infected erythrocyte surface antigen (RESA) contribute to the formation and maintenance of the knob structure. All these molecules form direct interactions with human erythrocyte cytoskeleton proteins (ECP)^3,4^.

ECPs are therefore central for the life cycle and proliferation of *P*. *falciparum*. In fact, alterations of the RBC cytoskeleton due to human genetic disorders such as hereditary spherocytosis (HS) and hereditary elliptocytosis (HE) (see Table 1 for associated gene defects) decrease the growth of *P*. *falciparum* in RBCs *in vitro*^9–11^. Similarly, Southeast Asian ovalocytosis (SAO), which is due to a heterozygous 27 bp deletion in the *SLC4A1* gene (encoding the band 3 protein), is associated with protection from cerebral *P*. *falciparum* malaria^12,13^. However, SAO also represents a protective factor for *P*. *vivax* infection and for *P*. *vivax* malaria severity^14^. Likewise, mice carrying mutations in genes encoding ECPs are protected from rodent malaria (*P*. *chabaudi*, *P*. *berghei*, *or P*. *yoelii* infection)^15–20^. Thus, genetic variants in genes encoding ECPs can affect the infection success or pathogenesis of malaria caused by *Plasmodium* parasites other than *P*. *falciparum*.

**Table 1:** Likelihood ratio test (LRT) statistics for F3x4 and F61 models in primate phylogenies.

Gene (Protein)	Human Disorder^a^	N° species	Model	M1a vs M2a^b^		M7 vs M8^b^		% negatively selected sites^d^	Positively selected sites^e^
				−2ΔlnL^c^	p value	−2ΔlnL^c^	p value		
***ACTB*** (Actin beta)		23							
			F3x4	0.004	0.998	0.004	0.998	35.47	na
			F61	0.004	0.998	0.0003	0.999		
***ADD1*** (Adducin 1)		23							
			F3x4	0	1	0.22	0.898	34.19	na
			F61	0	1	0.02	0.989		
***ADD2*** (Adducin 2)		23							
			F3x4	0.08	0.959	1.68	0.433	33.20	na
			F61	0	1	0.003	0.999		
***ANK1*** (Ankyrin-1)	HS	22							
			F3x4	0	1	19.03	7.39 × 10^−5^	40.67	na
			F61	5.47	0.07	23.33	8.57 × 10^−6^		
***DMTN*** (Dematin)									
			F3x4	0	1	2.59	0.27	26,67	na
			F61	0	1	2.97	0.23		
***EPB41*** (Protein 4.1)	**HE**	23							
**Reg1 (241 aa)**			F3x4	0.009	0.99	0.83	0.660	8.68	na
			F61	0.006	0.99	0	1		
**Reg2 (55 aa)**			F3x4	3.57	0.17	5.14	0.077	9.09	na
			F61	3.17	0.21	4.17	0.124		
**Reg3 (568 aa)**			F3x4	0.001	0.99	0	1	17.96	na
			F61	0	1	0	1		
***EPB42*** (Protein 4.2)	**HS**	23							
			F3x4	40.25	1.82 × 10^−9^	53.78	2.10 × 10^−12^	12.62	R9, P24, I102, R117, L159, Q163, R224, R243, F251, R289, L390, E487, R495, T501, H562, I572, N581, E675
			F61	31.63	1.35 × 10^−7^	37.80	6.19 × 10^−9^		
***MPP1*** (p55)		24							
			F3x4	0.003	0.999	0.009	0.995	32.83	na
			F61	0	1	0.011	0.994		
***RHAG*** (Ammonium transporter Rh type A)	**OHS**	24							
			F3x4	4.12	0.127	9.37	0.009	13.17	na
			F61	2.52	0.283	6.08	0.048		
***SLC4A1*** (Band 3)	**HS**, **SAO**	31							
			F3x4	6.43	0.04	29.02	5.01 × 10^−7^	32.69	E28, R112, E152, D235, H309, E658
			F61	15.76	3.79 × 10^−4^	32.44	9.02 × 10^−8^		
***SPTA1*** (α spectrin)	**HE**, **HS**	22							
			F3x4	30.43	2.47 × 10^−7^	55.68	8.12 × 10^−13^	26.50	E117, L148, V164, D430, Q434, T459, D466, I745, V1233, Q1332, Q1584
			F61	18.80	8.27 × 10^−5^	38.73	3.90 × 10^−9^		
***SPTB*** (β spectrin)	**HE**, **HS**	24							
			F3x4	0	1	17.94	1.27 × 10^−4^	42.69	na
			F61	0	1	2.90	0.234		
***TMOD1*** (Tropomodulin-1)		24							
			F3x4	0	1	0.02	0.991	20.89	na
			F61	0	1	0	1		
***TPM1*** (Tropomyosin alpha-1 chain)		23							
			F3x4	0	1	0.05	0.974	10.92	na
			F61	0	1	0.16	0.922		
***TPM3*** (Tropomyosin alpha-1 chain)		24							
**Reg1 (80 aa)**			F3x4	0	1	0	1	15.00	na
			F61	0	1	0	1		
**Reg2 (205 aa)**			F3x4	4.22	0.121	4.26	0.119	10.24	na
			F61	0.68	0.711	0.80	0.671		

Notes: ^a^Human red cell membrane disorders associated with ECP (https://www.ncbi.nlm.nih.gov/medgen/): HS, Hereditary spherocytosis; HE, hereditary elliptocytosis; OHS, overhydratate hereditary stomatocytosis; SAO, Southeast Asian ovalocytosis; ^b^M1a is a nearly neutral model that assumes one ω class between 0 and 1 and one class with ω = 1; M2a (positive selection model) is the same as M1a plus an extra class of ω > 1; M7 is a null model that assumes that 0 < ω < 1 is beta distributed among sites; M8 (positive selection model) is the same as M7 but also includes an extra category of sites with ω > 1; ^c^2ΔlnL: twice the difference of the natural logs of the maximum likelihood of the models being compared; ^d^Percentage of sites evolving under negative selection by FUBAR; ^e^Positions refer to the human sequence.

HE reaches frequencies up to 2% in African populations who live in malaria-endemic areas^21^ and SAO is very common in *P*. *falciparum*-endemic regions of Island Southeast Asia and Melanesia^22^. These observations clearly underscore the extremely strong selective pressure that *Plasmodium* parasites exerted on human populations^23^. Such selection most likely operated on a large number of human loci, including polymorphic variants in genes encoding ECPs^24^.

Several non-human primates (NHPs) are infected by *Plasmodium* parasites, and close relatives of *P*. *falciparum* and *P*. *vivax* were described in recent years in African great apes^25^. Indeed, *P*. *falciparum*, which belongs to the *Laverania* subgenus, originated from a single gorilla to human cross-species transmission event^26^. These observations and the notion whereby primate *Plasmodium* species diverged millions of years ago^27^ suggest that these parasites have exerted a selective pressure which was not limited to the recent history of human populations, but extended during the timing of primate (and possibly mammalian) speciation^27^. Herein, we thus investigate the evolutionary history of genes encoding ECPs in primates. We use different strategies to infer which parasites exerted the strongest selective pressure and to identify regions and sites that evolved in response to such pressure.

## Results

### Positive selection at erythrocyte cytoskeleton proteins

We first aimed to comprehensively analyze the selective pressure acting on primate genes that encode erythrocyte cytoskeleton proteins (ECP). In particular, we focused our attention on genes encoding ECPs that are involved in the remodeling of RBC during *Plasmodium* infection^3,4^. Some of these genes (*ANK1*, *EPB41*, *EPB42*, *SLC4A1*, *SPTA1*, and *SPTB*), when mutated, cause red cell membrane disorders (HE, HS, or SAO)^21^ (Table 1) and encode proteins that are directly bound by malaria parasite proteins.

Coding sequences were obtained for at least 22 primate species (Supplementary Table S1). These sequence data allow sufficient power to detect positive selection at primate genes^28^. Because recombination can be mistaken as positive selection^29,30^, DNA alignments were screened for the presence of recombination signals. *EPB41* and *TPM3* showed 3 and 1 recombination breakpoints, respectively; alignments were thus split in three and two regions (Table 1).

Pervasive positive selection was searched for using the “site models” implemented in the *codeml* program^31^. Using likelihood ratio tests (LRTs), c*odeml* compares models of gene evolution that allow (models M2a and M8) or disallow (models M1a and M7) a class of codons to evolve with dN/dS > 1. Thus, M2a and M8 represent the positive selection models that are tested against the neutral M1a and M7 models. The latter were rejected in favor of the positive selection models for *SLC4A1*, *SPTA1*, and *EPB42* (Table 1).

We next applied the Bayes Empirical Bayes (BEB) analysis, as well as the FUBAR and FEL methods (see Materials and Methods), to identify specific sites targeted by positive selection in these 3 genes. We applied a conservative strategy and called a site as positively selected only if it was detected by at least two methods.

**Figure 1:**
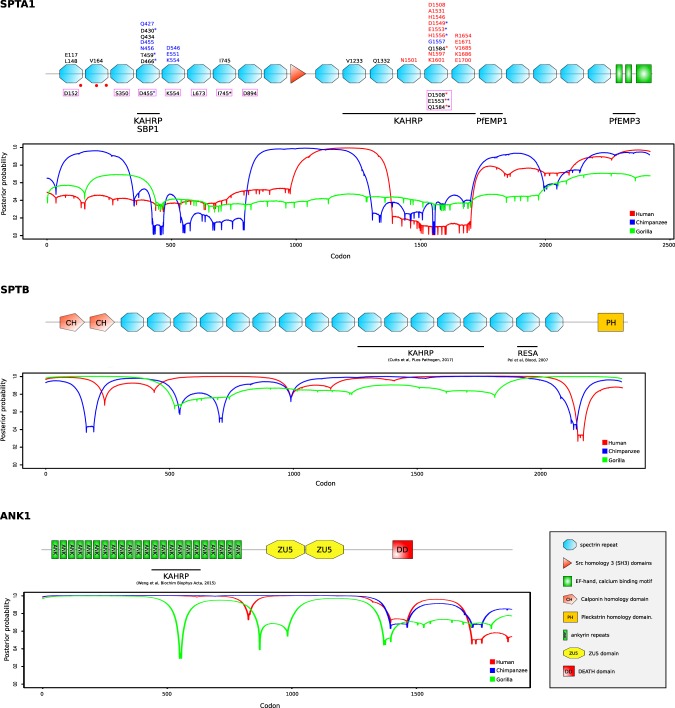
Domain representation of SPTA1, SPTB, and ANK1 proteins. Positively selected sites identified in the primate phylogeny (black), in Homininae lineages (human in red; chimpanzee in blue), as well as sites showing evolutionary rate shifts associated to the *Laverania* host state (magenta box) are reported. Mutations that cause hereditary elliptocytosis (HE) and decrease *P*. *falciparum* growth *in vitro* (red dots) and regions corresponding to the binding sites of *Laverania*-encoded proteins are also indicated. Asterisks indicate sites that were identified in different analyses (red asterisk: human lineage, blue asterisk: chimpanzee lineage, black asterisk: primate phylogeny). Plots represent the distribution of negatively selected codons across SPTA1, SPTB1, and ANK1 in Homininae lineages. In particular, the gammaMap-derived posterior probability of γ < 0 is plotted.

Several positively selected sites were detected in SPTA1, SLC4A1, and EPB42 (Table 1). In SPTA1, the 11 positively selected sites are distributed along the protein sequence, although four of them localize to the α4 spectrin repeat (Fig. 1), which is involved in direct interaction with KAHRP and SBP1 (skeleton binding protein 1) of *P*. *falciparum*^7,32^ (Fig. 1).

For SLC4A1, 5 out 6 positively selected sites localized to the cytosolic N-terminal domain, which is important as an anchoring point for erythroid cytoskeletal proteins (e.g. ankirin-1, spectrins, protein 4.1 and 4.2), denatured hemoglobin, and glycolitic enzymes^33^. The other positively selected site (E658) is located in the fourth extracellular loop and is required for band 3 association to glycophorin A (GPA)^34,35^. Both band 3 and GPA interact with *P*. *falciparum* merozoite surface protein 1 (MSP1) and band 3 is also bound by multiple *P*. *vivax* merozoite proteins^15,36,37^. Although the regions of band 3 involved in the interaction with merozoite proteins are located on extracellular loops different from loop 4^36,37^, E658 may modulate RBC invasion by (de)stabilizing the GPA-band 3 complex. In this respect it is worth noting that mouse models genetically deficient for band 3, and consequently lacking the GPA-band 3-protein 4.2 complex, are fully resistant to *P*. *yoelii*^15^.

Finally, in EPB42 the 18 positively selected sites are distributed along the protein sequence and one of them (E487) falls in the region involved in the interaction with α spectrin^38^. Unfortunately, the details of the interaction between EPB42 and *Plasmodium*-encoded proteins are unknown.

**Figure 2:**
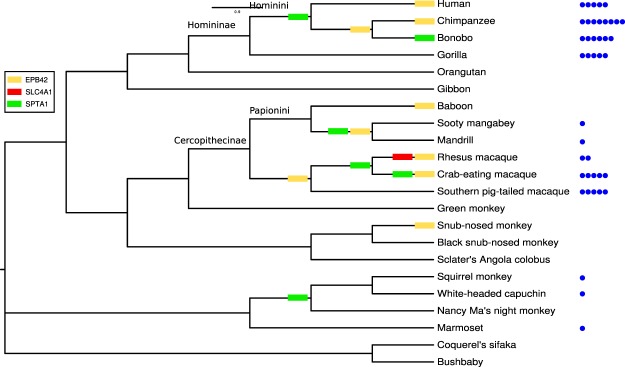
Free ratio model analysis. Results are shown for a restricted primate phylogeny, which includes only species with available sequence information for *EPB41*, *SLC4A1*, and *SPTA1* genes. Branches showing dN/dS > 1 are marked with colored rectangles. The number of different *Plasmodium* species identified in each primate species are represented with blue circles.

To explore possible variations in selective pressure across the primate phylogeny for the three positively selected genes (*EPB42*, *SLC4A1*, and *SPTA1*), we applied the free ratio (FR) model implemented in the PAML software. In particular, we tested whether models that allow dN/dS to vary along branches had significant better fit to the data than models that assume one same dN/dS across the entire phylogeny^39^. Results indicated that the FR model fitted the data better than the null model for all three positively selected genes (data not shown), suggesting that the selection acted differently across the phylogeny, with some branches showing *dN/dS* values higher than 1. We then overlaid the selection signals over the phylogenetic tree to obtain a glimpse of whether positive selection acted on specific lineages (Fig. 2). Most of the Homininae and Cercopithecinae branches showed at least one gene with dN/dS > 1, and this is particularly abundant at the internal and external branches of the Hominini and Papionini tribes (Fig. 2). We next retrieved information on *Plasmodium* infection for the taxa represented in the phylogeny^40–42^ (Supplementary Table S1). Notably, episodes of positive selection tend to be more common for species that host a larger number of different *Plasmodium* parasites (Fig. 2).

### *Laverania*-driven selection at *SPTA1*

To investigate whether some of the selective events we identified in genes encoding ECPs were due to the pressure imposed by *Laverania*, we used a recently developed approach (TraitRateProp) that allows testing whether a proportion of sites in a gene or region exhibit evolutionary rate shifts that are associated with the state of a binary phenotypic trait^43^. We thus set phenotype states for species that are or that are not natural hosts for *Laverania* (see Materials and Methods). Strong evidence that the rate of sequence evolution is associated with *Laverania* infection was obtained for *SPTA1* alone (chi-squared likelihood ratio test, *p value* = 1.84 × 10^−3^; relative rate = 10). In particular, TraitRateProp identified 10 SPTA1 residues that show evolutionary rate shifts (Bayes-factor >= 8) associated to the *Laverania* host state (Fig. 1). Two of these sites (residues I745 and Q1584) were also identified in the codeml analysis described above.

### Positive selection in Homininae lineages

To further explore the selective pattern in species that are hosts for *Laverania*, we took advantage of the availability of genetic diversity data for humans and great apes^44,45^ to search for positively selected sites in the human, chimpanzee, and gorilla lineages. Specifically, we used a population genetics-phylogenetics approach (gammaMap^46^) which leverages information of intra-species polymorphism and between-species divergence. This approach has higher power than those described above for selective events that occurred during the most recent evolutionary history of specific lineages.

The gammaMap method categorizes codon-wise population-scaled selection coefficients (γ) into different classes, ranging from strongly beneficial (γ = 100) to inviable (γ = −500), with γ equal to 0 indicating neutrality. We called positively selected sites as those having a cumulative probability higher than 0.8 of γ ≥ 1.

Several positively selected sites were identified in the human and chimpanzee lineages for *SPTA1*. In humans, positive selection also drove the evolution of a few codons in *EPB41*, *EPB42*, and *TMOD1* (Table 2). In *RHAG*, a few positively selected codons were detected in all three great ape lineages.

**Table 2:** Positively selected sites in great ape lineages.

Positively selected sites (posterior probability ≥ 0.80)^a^		
Human^b^	Chimpanzee^b^	Gorilla^b^
***EPB41***		
Q190, T195	—	—
***EPB42***		
Y74, **L159**, N172, K228	—	V539, G577, A583
***RHAG***		
V86, K407	K40, I46, D234,C237	T42, I82, T139, E199, R238, N395
***SLC4A1***		
—	A255, I262	—
***SPTA1***		
N1501, D1508, A1531, H1546, D1549, E1553, H1556, **Q1584**, N1597, K1601, R1654, E1671, V1685, K1686, E1700	Q427, **D430**, D455, N456, **T459**, **D466**, D546, E551, K554, D1549, E1553, H1556, G1557	—
***TMOD1***		
M285	I210	—

Note. ^a^Posterior probability of γ > 0 as detected by gammaMap. ^b^Positively selected site in both primate phylogeny and specific lineage are in bold. Positions refer to the human sequence.

In SPTA1, we observed that positively selected sites are clustered into two specific regions: the α4 and α13–15 spectrin repeats. Both regions were reported to directly interact with *P*. *falciparum* proteins^7,8^ (Fig. 1). Notably, all sites that were positively selected in the human lineage map to the distal KAHRP interaction domain, whereas the sites identified in the chimpanzee lineage localize to both KAHRP-binding regions. Five of the sites we identified with GammaMap were also identified by TraitRateProp (Fig. 1).

These data suggest that the selection signals identified in human and chimpanzee SPTA1 derive from an arms-race with KAHRP or, possibly, with SBP1.

### Strong selective constraints limit *ANK1* and *SPTB* evolution

β spectrin and ankirin-1 play very important role in stabilizing the erythrocyte membrane and they are bound by several *Laverania* proteins. For instance, KAHRP binds β spectrin repeats 10–14 with three-fold higher affinity compared to α spectrin 12–16 repeats^8^. Ankirin is also targeted by KAHRP^6^ and other *P*. *falciparum* proteins^47,48^. Indeed, a previous study that analyzed the evolution of genes that interact with *Apicomplexa* parasites indicated that *SPTB* and *ANK1* display features suggestive of adaptive evolution in mammals^49^. However, we detected no positive selection at *SPTB* and *ANK1*, either in the entire primate phylogeny or in Homininae lineages. We thus reasoned that this finding may result from strong functional constraints that prevent amino acid replacements to accrue in response to *Plasmodium*-exerted selective pressures. To test this possibility, we used FUBAR to calculate the percentage of sites that are target by negative selection in ECP-encoding genes. SPTB and ANK1 showed the highest portion of negatively selected sites (Table 1). We next explored the distribution of negatively selected codons across SPTB1 and ANK1 in Homininae by plotting the gammaMap-derived posterior probability of γ < 0. Results indicated that both genes display extensive regions of selective constraint in humans and great apes (chimpanzee and gorilla) and these regions cover the binding sites of *Laverania*-encoded proteins (Fig. 1).

**Figure 3:**
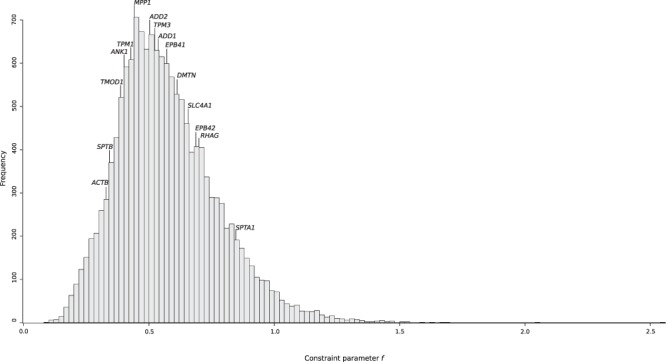
Selective constraints at ECP-encoding genes in human populations. The distribution of the constraint parameter *f* calculated for reference genes and ECP-encoding genes is reported.

To assess whether *SPTB* and *ANK1* were also severely constrained during to the more recent evolution of human populations, we used SnIPRE, which contrasts polymorphism and divergence data at nonsynonymous and synonymous sites, to calculate the constraint parameter *f*. *f* represents the proportion of non-synonymous mutations that are tolerated and, therefore, low values of *f* indicate strong constraints^50^. *f* values were calculated for 14881 human genes and a distribution was obtained (Fig. 3). *SPTB* and *ANK1* displayed some of the lowest *f* values among ECP-encoding genes and their *f* values were well below the median for all human genes (Fig. 3). Conversely, *SPTA1* showed the weakest selective constraint among ECP-encoding genes.

Overall, these results suggest that functional constraint prevent SPTB and ANK1 to evolve in response to the selective pressure exerted by *Plasmodium* proteins that remodel the RBC cytoskeleton.

### Positive selection at *Laverania* genes that encode proteins involved in RBC remodeling

Several *Laverania* proteins interact with components of the erythrocyte cytoskeleton. Among these the best known are PfEMP1, MESA, PfEMP3, PHIST proteins, RESA, and KAHRP^3^. PfEMP1 is encoded by several *var* genes, and a number of PHIST proteins were detected in *Laverania*^3,51^. These proteins cannot thus be analyzed using molecular evolution methods based on ortholog identification and alignment. Similar considerations apply to RESA proteins, which are encoded by at least three paralogs^51^. We thus focused on KAHRP (*PF3D7*_*0202000*), MESA (*PF3D7*_*0500800*), and EMP3 (*PF3D7*_*0201900)*. MESA and EMP3 are composed of unique N-terminal regions and extensive repetitive sequences in the central and C-terminal portions. Repetitive regions could not be reliably aligned and the analysis was thus restricted to the N-terminal portions, which harbor motifs responsible for binding to ECPs^52–54^.

Alignments of KAHRP and the N-terminal regions of EMP3 and MESA included available *Plasmodium falciparum* strains, as well as the sequences of *P*. *reichenowi* and *P*. *gaboni* (this latter was not available for MESA) (Supplementary Table S2). Evidence of positive selection was searched for using the codeml LRT tests, as described above.

**Figure 4:**
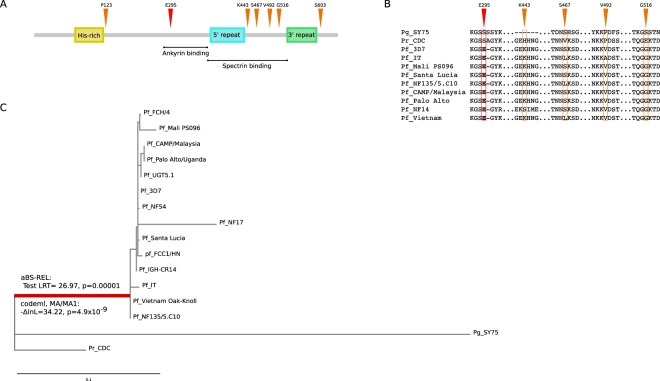
Positive selection at *Laverania KAHRP* genes. (**A**) Schematic representation of KAHRP domains. Sites targeted by pervasive positive selection in the *Plasmodium* phylogeny (orange) and by episodic positive selection on the branch leading to *P*. *falciparum* (red) are shown. Regions corresponding to the binding sites of ankiryn-1 and spectrin proteins are indicated. (**B**) Protein alignments of representative *Plasmodium* strains for the regions surrounding positively selected sites. (**C**) Phylogenetic tree of *Laverania KAHRP*. The branch leading to *P*. *falciparum* strains and showing evidence of episodic positive selection is in red. The results of codeml and aBS-REL are also reported.

The neutral model was not rejected in favor of the positive selection models for *EMP3*. Conversely, models that allow a class of codons to evolve with dN/dS > 1 fitted the data better than the neutral model for *KAHRP* and *MESA* (Table 3). Notably, four of the six positively selected sites in KAHRP are located in a domain which binds α and β spectrins^8^. Some of these sites are variable across *Laverania* species and among *Plasmodium falciparum* isolates (Fig. 4). In contrast, the only selected site in MESA was not located in the protein region that binds protein 4.1^52,53^.

**Table 3:** Likelihood ratio test (LRT) statistics for F3x4 and F61 models in *Plasmodium* phylogenies.

Gene	N° strain/isolates	Model	M1a vs M2a^a^		M7 vs M8^a^		Positively selected sites^c^
			−2ΔlnL^b^	p value	−2ΔlnL^b^	p value	
*KAHRP*	16						
		F3x4	34.81	2.78 × 10^−08^	35.78	1.70 × 10^−08^	P123, K443, S467, V492, G516, S603
		F61	36.05	1.92 × 10^−9^	37	1.18 × 10^−9^	
*MESA*	11						
		F3x4	13.54	1.15 × 10^−03^	13.71	1.05 × 10^−03^	N315
		F61	6.61	0.010	7.24	0.0071	
*EMP3*	15						
		F3x4	1.70	0.43	1.76	0.41	—
		F61	1.50	0.22	6.39	0.011	

Notes: ^a^M1a is a nearly neutral model that assumes one ω class between 0 and 1 and one class with  ω = 1; M2a (positive selection model) is the same as M1a plus an extra class of ω > 1; M7 is a null model that assumes that 0 < ω < 1 is beta distributed among sites; M8 (positive selection model) is the same as M7 but also includes an extra category of sites with ω > 1; ^b^2ΔlnL: twice the difference of the natural logs of the maximum likelihood of the models being compared; c. Positions refer to the Pf_3D7 strain sequence.

Finally, we assessed whether episodic positive selection acted on the internal branches of the KAHRP and MESA phylogenies. Evidence of selection was detected for the branch leading to *P*. *falciparum* strains for KAHRP (Fig. 4). Only one positively selected sites was detected and is located in a 79-amino acid region that is sufficient to bind ankirin^6^.

## Discussion

*Plasmodium* parasites represented one of the major selective pressures during the recent evolutionary history of human populations^23,24^ and, in endemic areas, malaria remains an important cause of death, especially for children and pregnant women (WHO, http://www.who.int/malaria/publications/world-malaria-report-2016/). Data from NHP populations in Africa and Asia indicated that *Plasmodium* infections are highly prevalent and that these animals can be simultaneously infected by multiple *Plasmodium* species^1,55–61^. Although *Plasmodium* infection is generally thought to result in no or very weak pathology in NHPs, recent reports have indicated that, as in humans, parasitemia is higher in young and pregnant chimpanzees^62–64^. In a young individual, transitory malaria-like symptoms were also described as a consequence of *P*. *reichenowi* infection^65^. Thus, *Plasmodium* parasites are potentially pathogenic for NHPs and these animals are likely to have evolved resistance mechanisms. In line with the idea that *Plasmodium* parasites have exerted a long-standing selective pressure on NHPs, host molecules that serve as the receptor for *P*. *falciparum* (basigin) and *P*. *vivax*/*P*. *knowlesi* (Duffy antigen) were previously shown to have evolved under positive selection in primate phylogenies^66,67^. It is thus conceivable that other genes that encode molecules involved in *Plasmodium* infection or pathogenesis have evolved to avoid or limit the fitness costs imposed on primates by these parasites. Indeed, a previous analysis with a limited number of primate species detected evidence of adaptive evolution at the *SLC4A1* gene and interpreted this in terms of *Laverania*-driven selection^68^. Herein, we detected evidence of pervasive positive selection at genes encoding α spectrin, protein 4.2 and, in agreement with Steiper *et al*.^68^, band 3.

Notably, we do not imply that *Plasmodium* parasites represented the only selective forces that acted on primate ECP-encoding genes. In fact, evidence of adaptive evolution at SPTA1 was recently reported in a study that focused on mammals and detected positively selected sites on several branches of the phylogeny, including species that are not known to be infected by *Plasmodium* parasites^49^. However, we analyzed several RBC genes and we detected the strongest evidence of positive selection for those that encode proteins directly interacting with *Plasmodium*-encoded components. Also, we found that episodes of positive selection tended to occur more frequently in primate species that host a larger number of *Plasmodium* parasites. Finally, in the case of SPTA1, some of the sites we identified are located close to mutations that were reported to cause HE and to decrease *P*. *falciparum* growth *in vitro*^9^ (Fig. 1). For this gene, TraitRateProp supported the view that a fraction of sites shifted their evolutionary rates in response to *Laverania*-exerted selective pressure. Some of these sites are located in the regions bound by PfKAHRP and/or by PfSBP1 and, indeed, they partially overlap with the selection signals we detected in the human and chimpanzee lineages. These latter signals were strongly clustered in the α 4–5 and α 14–15 spectrin domains, suggesting that the recent evolution of *SPTA1* was dominated in humans and chimpanzees by a selective pressure that targeted these domains. Overall, these observations suggest that malaria parasites contributed to the shaping of *SPTA1* genetic diversity in primates. Experimental validation will nonetheless be required to determine whether selected sites modulate the binding of *Plasmodium* proteins and affect parasite replication.

We note that some controversy exists about the binding site of PfKAHRP to human α spectrin. An initial analysis indicated an interaction with spectrin repeat α 4^7^, whereas a more recent study detected no binding with this region and indicated repeats α 16–17 (corresponding to α 15–16 spectrin domains in Fig. 1) as the interaction site with PfKAHRP^8^. Whereas additional experiments will be required to clarify the reasons for these discrepancies and whether they may derive from the use of different *P*. *falciparum* strains (IT and 3D7), we note that the α 4 spectrin repeat, which may not bind PfKAHRP and is nonetheless targeted by strong selection in chimpanzee, represents the interaction site with PfSBP1^32^. SBP1 orthologs are found in *P*. *reichenowi* and *P*. *gaboni*, suggesting that the selective pressure responsible for the observed selection signals at the chimpanzee α 4 is related to SBP1 binding. Indeed, remarkable differences are observed in the selection pattern of the three ape *SPTA1* genes. Whereas all selected sites in humans are located in α 13–15, selection in chimpanzees mainly targeted α 4–5, and no positively selected site was detected in gorillas. Unfortunately, the genomes of gorilla-infecting *Laverania* are unavailable and it is thus unknown whether they also encode KAHRP and SBP1. This is highly plausible, though, as *P*. *preafalciparum* and *P*. *blacklocki* are more closely related to *P*. *falciparum* and *P*. *reichenowi* than *P*. *gaboni*^25^, which encodes both proteins. The reasons why no selection signals were detected at the gorilla *SPTA1* gene remain to be investigated. We nonetheless found positively selected sites in the gorilla protein 4.1 region that is bound by SBP1^32^. Overall, these results suggest that the interactions between *Laverania*-encoded proteins that are exported to the erythrocyte membrane and ECPs are dynamic and possibly change over time or during parasite speciation events.

Data herein also clearly indicate that the ability of primate hosts to adapt in response to *Plasmodium*-exerted pressure is limited by functional constraints. Despite the high-affinity binding of PfKAHRP to β spectrin^8^, no signal of selection was detected at primate *SPTB* genes. Across the different time frames considered herein, namely primate radiation, great ape speciation and human evolution, *SPTB* and, to a lesser extent *ANK1*, showed extensive signatures of purifying selection, suggesting that most amino acid replacements cause substantial fitness loss. From the parasite’s perspective these molecules most likely represent ideal interactors, as they are not allowed to engage in molecular arms-races.

A limitation of this study is that the evolutionary analyses of *Plasmodium* genes were conducted using a very limited number of orthologs, as few genome sequences of *Laverania* species are available. Under these circumstances, the power to detect selection is limited, although false positives are not expected^69^. This might explain why signals of selection were not detected for EMP3 and only one selected site was identified in MESA. We also mention that, as many proteins encoded by *Plasmodium* parasites, those we analyzed herein contain repetitive sequences^70^. Whereas highly repetitive regions must be filtered out to allow reliable alignments, they most likely contribute to protein evolution and parasite adaptation by regulating protein localization, binding affinities, and structural properties^70^. Thus, analyses herein necessarily fail to account for an important source of variability that may contribute to modulate the binding of *Plasmodium* proteins to the erythrocyte cytoskeleton. Despite these limitations, we were able to identify some selected sites in KAHRP, most of which are located in the spectrin-binding domain^8^.

KAHRP-like proteins were described in several *Plasmodium* species although it is unclear whether they represent orthologs of PfKAHRP as these proteins display different domain structures^71^. In fact, we used PSI-BLAST to search for proteins similar to PfKAHRP and we detected significant homology over a considerable protein length for *Laverania* species only. The only exception was a protein encoded by *Plasmodium* fragile (GenBank: CAB96390.3) which showed almost 98% identity to *Plasmodium falciparum* KAHRP and was more closely related to PfKAHRP than PrKAHRP or PgKAHRP. This high level of identity was previously noted^51^. However, because *P*. *fragile* belongs to a different *Plasmodium* subgenus than *Laverania*, such high identity is surprising and the *P*. *fragile* protein was never characterized in a published manuscript. Thus, this sequence was not included in our analyses (that focuses on *Laverania*) and we suggest that caution should be used when inferring its real occurrence in *P*. *fragile*.

In summary, data herein indicate that SPTA1 and KAHRP have been engaged in a genetic conflict and that band 3 may also have evolved in response to the selective pressure exerted by *Plasmodium* parasites. Conversely, other ECPs have been strongly constrained throughout primate evolutionary history, limiting their ability to accommodate changes that may confer resistance to *Plasmodium* infection.

## Materials and Methods

### Evolutionary analysis in Primates and Laverania phylogenies

Coding sequence information for primate species were retrieved from the NCBI database (http://www.ncbi.nlm.nih.gov/) and from UCSC server (http://genome.ucsc.edu/*)*. A complete list of species analyzed for each gene is reported in Supplementary Table S1. Sequence alignments were performed using the RevTrans 2.0 utility^72^.

For Laverania genes (*KAHRP*, *MESA*, and *EMP3*), coding sequences were retrieved from the NCBI database (http://www.ncbi.nlm.nih.gov/) or via interrogation of the European Nucleotide Archive (https://www.ebi.ac.uk/ena) using the protein ID accession. Lists of accession numbers are reported in Supplementary Table S2.

PSI-BLAST was run via the EMBL-EBI dedicated website (https://www.ebi.ac.uk/Tools/sss/psiblast/).

We used MAFFT^73^ to generate multiple sequence alignments and GUIDANCE2^74^ for filtering unreliably aligned codons with a score <0.90^75^.

Each alignment was screened for the presence of recombination breakpoints using GARD (Genetic Algorithm Recombination Detection)^76^, a program that uses phylogenetic incongruence among segments of a sequence alignment to detect the best-fit number and location of recombination breakpoints. Evidence of recombination was detected for *EPB41* and *TPM3*, whereas no breakpoints were detected for all the remaining primate genes and for *Laverania* genes.

To detect positive selection, we used the site models implemented in PAML^31^ for whole gene alignments or independently for sub-regions defined in accordance with the recombination breakpoints. Specifically, we fitted site models that allow (M2a, M8) or disallow (M1a, M7) a class of sites to evolve with ω > 1 to the data using the F3x4 and the F61 codon frequency models. Statistical significance was assessed by comparing twice the ΔlnL of the two models with a χ^2^ distribution with 2 degrees of freedom. We considered a gene to be positively selected if both comparisons, M1a vs M2a and M7 vs M8, were statistically significant for both codon frequency models (F3x4 and F61). Input phylogenetic trees were reconstructed using the phyML program with a maximum-likelihood approach, a General Time Reversible (GTR) model plus gamma-distributed rates and 4 substitution rate categories^77^.

Positively selected sites were identified using the codeml Bayes Empirical Bayes analysis (BEB, from model M8 with a cutoff of 0.90)^78^, the Fixed effects likelihood (FEL, with a default cutoff of 0.1)^79^, and the Fast Unconstrained Bayesian AppRoximation (FUBAR, with a default cutoff of 0.90)^80^. To limit false positives, we considered a site as positively selected if it was detected by at least two different methods.

To analyze the pattern of selection at *EPB42*, *SLC4A1*, and *SPTA1* genes across the primate phylogeny we applied the free ratio (FR) model implemented in the PAML software^39^. In particular, the FR model was used to estimate the value of dN/dS (non-synonymous substitution/synonymous substitution rate ratio) for each branch of the phylogenies and was compared with a null model that estimates one dN/dS for the entire phylogeny. Statistical significance is assessed by comparing twice the ΔlnL of the two models with a χ^2^ distribution with degrees of freedom equal to the difference in model parameters.

Data on *Plasmodium* distribution in NHP were obtained from a previous work that used published records of *Plasmodium* parasites in NHPs to provide a global overview of primate malarias^1^. These data were updated through literature searches of studies published after 2005^40–42^.

In order to identify specific branches with a proportion of sites evolving with dN/dS > 1 in the *Plasmodium KAHRP* and *MESA* phylogenies, we used the adaptive Branch-Site Random Effects Likelihood method (aBS-REL)^81^. This method applies sequential likelihood ratio tests to identify branches under positive selection without a priori knowledge about which lineages are of interest^81^; branches identified using this approach were cross-validated using the branch-site likelihood ratio tests from PAML (models MA and MA1). To identified sites evolving under positive selection on specific branches we used the BEB analysis from MA (with a cutoff of 0.90) and the Mixed Effects Model of Evolution (MEME) (with the default cutoff of 0.1)^82^. MEME allows the distribution of ω to vary from site to site and from branch to branch at a site. Again, to limit false positives, only sites confirmed by both methods were considered as positively selected.

GARD, FEL, FUBAR, MEME, and aBS-REL analyses were performed either through the DataMonkey server^83^ (http://www.datamonkey.org) or run locally (through HyPhy^84^).

The name and the localization of the domains in the protein sequences are taken from Uniprot^85^.

### Population genetics-phylogenetics analysis in Homininae

In order to study the evolution in Homininae and to gain insight into the more recent selective events in specific lineages (human, chimpanzee, and gorilla lineages), we applied a population genetics-phylogenetics approach (gammaMap^46^) for *ANK1*, *EPB41*, *EPB42*, *SLC4A1*, *SPTA1*, and *SPTB* genes. Ancestral sequences were reconstructed by parsimony from the human, chimpanzee, gorilla, orangutan, gibbon, and macaque sequences.

For human analyses, genotype data from the Phase 1 of the 1000 Genomes Project were retrieved from the dedicated website *(*http://www.1000genomes.org/*)*^45^; in particular, SNP information were retrieved for individuals of three human populations: African (Yoruba), European, and East Asian (Chinese). For the chimpanzee and gorilla analyses, we used SNP information from 25 and 27 individuals, respectively^44^.

gammaMap uses intra-specific variation and inter-specific diversity to estimate the distribution of population-scaled selection coefficients (γ) along coding regions. The program classifies γ values into 12 categories, ranging from strongly beneficial (γ = 100) to inviable (γ = −500), with γ equal to 0 indicating neutrality. In the analysis, we assumed θ (neutral mutation rate per site), k (transitions/transversions ratio), and T (branch length) to vary among the gene following log-normal distributions. For p (the probability that adjacent codons share the same population-scaled selection coefficient) we assumed a uniform distribution. We set the neutral frequencies of non-STOP codons to 1/61. For population-scaled selection coefficients we considered a uniform Dirichlet distribution with the same prior weight for each selection class. For each gene, two Markov Chain Monte Carlo runs of 100,000 iterations each were run with a thinning interval of 10 iterations. Runs were compared to assess convergence and merged to obtain posterior probabilities. To be conservative, we declared a codon to be targeted by positive selection when the cumulative posterior probability of γ ≥ 1 was ≥0.80.

### Purifying selection in humans

The strength of purifying selection was estimated using SnIPRE^50^, a tool that relies on the comparison of polymorphism and divergence data from synonymous and non-synonymous sites within genes. SnIPRE uses a generalized linear mixed model to represent the genome-wide variability among categories of mutations and to estimate its functional consequence. We estimated the degree of selective constraints at each gene using the *f* parameter, which is the proportion of non-synonymous mutation that are not deleterious.

The *f* parameter was estimated for each gene and for 14881 autosomal coding human genes used as reference.

SNP information were retrieved for individuals of all 1000 Genomes Project Phase 1 populations^45^. To evaluate divergence within genes, we used the liftOver tool to convert human GRCh37/hg19 genome coordinates to *Pan troglodytes* (CGSC 2.1.3/PanTro3) coordinates; we selected only genes that could be mapped onto chimpanzee genome (n = 14805).

## Electronic supplementary material

Supplementary Tables S1 and S2
