# Supplementary material for: Genetic conflicts with Plasmodium parasites and functional constraints shape the evolution of erythrocyte cytoskeletal proteins
Source: Sci Rep. 2018 Oct 2;8:14682. doi: 10.1038/s41598-018-33049-y (PMC6168477; doi:10.1038/s41598-018-33049-y)
Supplement: Supplementary file 1 — Supplementary Tables S1 and S2 [file 41598_2018_33049_MOESM1_ESM.pdf]

Genetic conflicts with *Plasmodium* parasites and functional constraints shape the evolution of erythrocyte cytoskeletal proteins

Manuela Sironi[1] , Diego Forni[1] , Mario Clerici[2,3] , Rachele Cagliani[1*]

[1] Bioinformatics, Scientific Institute, IRCCS E. Medea, 23842 Bosisio Parini, Lecco, Italy.

[2] Department of Physiopathology and Transplantation, University of Milan, 20090 Milan, Italy.

[3] Don C. Gnocchi Foundation ONLUS, IRCCS, 20148 Milan, Italy.

Supplementary Table S1. List of primate species and their *Plasmodium* parasites

Scientific name	**Common name**	***ACTB***	***ADD1***	***ADD2***	***ANK1***	***DMTN***	***EPB41***	***EPB42***	***MPP1***	***RHAG***	***SLC4A1***	***SPTA1***	***SPTB***	***TMOD1***	***TPM1***	***TPM3***	***Known malaria parasites***
Aotus nancymaae *	Nancy Ma's night monkey																
Callicebus moloch	Red-bellied titi																
Callithrix jacchus *	Marmoset																*P. brasilianum*
Carlito syrichta/Tarsier syrichta	Philippine tarsier																
Cebus capucinus *	White-headed capuchin																*P. brasilianum*
Cercocebus atys *	Sooty mangabey																*P. gonderi*
Chlorocebus aethiops	Grivet/Vervet monkey																
Chlorocebus sabaeus *	Green monkey																
Colobus angolensis *	Sclater's Angola colobus																
Colobus guereza	Mantled guereza																
Gorilla gorilla *	Gorilla																*P. adleri, P. blacklocki, P. falciparum, P. schwetzi, P. vivax*
Homo sapiens *	Human																*P. falciparum, P. vivax, P. malariae, P. knowlesi, P. ovale*
Lagothrix lagotricha	Woolly monkey																
Lemur catta	Ring-tailed lemur																
Macaca fascicularis *	Crab-eating macaque																*P. coatneyi, P. cynomolgi, P. fieldi, P. inui, P. knowlesi*
Macaca mulatta *	Rhesus macaque																*P. cynomolgi, P. inui*
Macaca nemestrina *	Southern pig-tailed macaque																*P. coatneyi, P. cynomolgi, P. fieldi, P. inui, P. knowlesi*
Mandrillus leucophaeus *	Mandrill																*P. gonderi*
Microcebus murinus	Mouse lemur																
Nomascus Gabriellae	Yellow-cheeked gibbon																
Nomascus leucogenys *	Gibbon																
Otolemur garnettii *	Bushbaby																
Pan paniscus *	Bonobo																*P. falciparum, P. gaboni, P. ovale curtisi, P. lomamiensis, P. malariae-related, P. vivax*
Pan troglodytes *	Chimpanzee																*P. billcollinsi, P. falciparum, P. gaboni, P. malariae, P. ovale, P. reichenowi. P. schwetzi. P. vivax*
Papio hamadryas *	Baboon																
Pongo abelii *	Orangutan																

Propithecus coquereli *	Coquerel's sifaka																
Rhinopithecus bieti *	Black snub-nosed monkey																
Rhinopithecus roxellana *	Snub-nosed monkey																
Saguinus labiatus	White-lipped tamarin																
Saimiri boliviensis boliviensis *	Squirrel monkey																*P. brasilianum*

Note. Species marked with asterisks were included in the restricted primate phylogeny shown in Figure 2. Known malaria parasites are reported only for primates marked with asterisks.

Supplementary Table S2. List of *Plasmodium* strains/isolates.

		Strain/Isolate	Nucleotide ID	Protein ID used to interrogate ENA
KAHRP	*P. falciparum*	Pf_3D7	XM_001349498	
		Pf_CAMP/Malaysia	KI927677	
		Pf_FCC1/HN	AF275687	
		Pf_FCH/4	KI927792	
		Pf_IGH-CR14	GG665204	
		Pf_IT	PFIT_0201300	
		Pf_Mali PS096	ETW51738	
		Pf_NF135/5.C10	KI926017	
		Pf_NF17	Y00060	
		Pf_NF54	KE123721	
		Pf_Palo Alto/Uganda	KI927240	
		Pf_Santa Lucia	EUT93034	
		Pf_UGT5.1	KE124379	
		Pf_Vietnam Oak-Knoll	KI925011	
	P. gaboni	Pg_SY75	LVLB01000003	
	P. reichenowi	Pr_CDC	HG810763	
MESA	*P. falciparum*	Pf_3D7	XM_001351531	
		Pf_Dd2	DS016123	KOB85353
		Pf_FCH/4	KI928169	ETW26829
		Pf_HB3	CH672045	KOB62125
		Pf_IGH-CR14	GG665375	KNG77757
		Pf_IT	PFIT_0500900	
		Pf_Palo Alto/Uganda	AF056936	
		Pf_RAJ116	GG664386	KNC37107
		Pf_Santa Lucia	KE123479	EUT91020
		Pf_UGT5.1	KE124768	EWC73405
	P. reichenowi	Pr_CDC	HG810766	
EMP3	*P. falciparum*	Pf_3D7	XM_001349497	
		Pf_7GB	KE123587	EUR79230
		Pf_CAMP/Malaysia	KI927459	ETW63921
		Pf_IGH-CR14	GG665204	KNG76792
		Pf_IT	PFIT_0201200	
		Pf_MaliPS096_E11	KI925479	ETW51737
		Pf_MC	AH008249	
		Pf_NF135/5.C10	KI926017	ETW45260
		Pf_NF54	KE123721	EWC90929
		Pf_Palo Alto/Uganda	KI927240	ETW57704
		Pf_Santa Lucia	KE123473	EUT93033
		Pf_Tanzania (2000708)	KI926269	ETW39099
		Pf_UGT5.1	KE124379	EWC79156
	P. gaboni	Pg_SY75	XM_018783133	
	P. reichenowi	Pr_CDC	HG810763	

Note. ENA: European Nucleotide Archive.
